# Canadian real-world study of access and clinical results using dupilumab for chronic rhinosinusitis with polyps

**DOI:** 10.1186/s40463-022-00570-0

**Published:** 2022-04-25

**Authors:** Shaun J. Kilty, Andrea Lasso

**Affiliations:** Department of Otolaryngology-Head and Neck Surgery, University of Ottawa, The Ottawa Hospital, 737 Parkdale Ave., Room 259, Ottawa, ON K1Y 1J8 Canada

**Keywords:** Monoclonal antibody, Dupilumab, Chronic rhinosinusitis, Nasal polyps

## Abstract

**Background:**

Dupilumab is the first monoclonal antibody therapy to be approved in Canada for the treatment of Chronic Rhinosinusitis with Nasal Polyps (CRSwNP). The goal of the study was to assess its effectiveness and efficacy in a real-world setting. This study aims to assess how clinical outcomes of biologic therapy in real-world application (effectiveness) correspond to outcomes in clinical trials (efficacy) and to look into factors that might explain an efficacy-effectiveness gap.

**Methods:**

A retrospective study evaluating disease specific sinonasal outcomes routinely collected for clinical care. This study included patients who were evaluated for coverage of dupilumab at a tertiary care rhinology clinic for the treatment of CRSwNP in the first year since dupilumab was approved in Canada for this indication. Sinonasal outcomes were be evaluated by collecting data on the Sino-Nasal Outcome Test (SNOT)-22 questionnaire.

**Results:**

Eighty-five patients were considered for dupilumab therapy during the study period, 49% patients were able to attain coverage for the requested therapy. The mean SNOT-22 score at baseline was 60.56 (SD 21.63). After 16 weeks of treatment the mean SNOT-22 score decreased by 37 points to 23.36; at 28 weeks the mean SNOT-22 was 23.47. After 1 year, the mean SNOT-22 score was 14.37.

**Conclusion:**

Patients treated with dupilumab for CRSwNP at out tertiary rhinology clinic showed substantial clinical symptom improvement that is similar to that observed in prior randomised clinical trials. No serious adverse effects related to dupilumab were reported in this cohort. Long-term follow-up is needed to inform effectiveness analyses beyond the 1 year clinical trial duration.

**Graphical Abstract:**

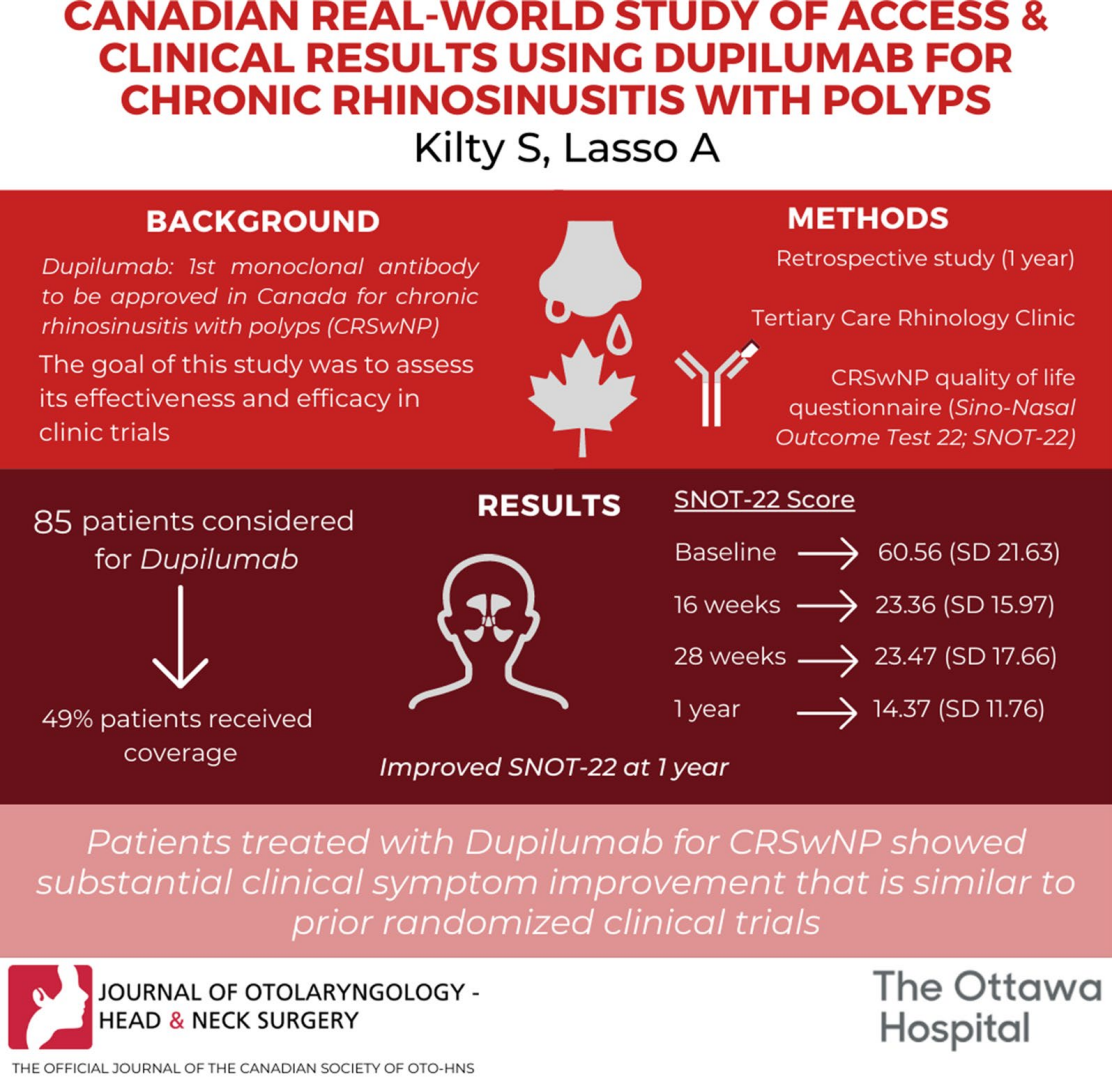

## Introduction

Chronic rhinosinusitis with polyps (CRSwNP) is a common idiopathic heterogeneous inflammatory disease of the nose and paranasal sinuses that is commonly characterized by the clinical symptoms of nasal obstruction, rhinorrhea and olfactory loss. The diagnosis of CRSwNP is dependent upon the presence of major symptoms for at least eight consecutive weeks with confirmatory findings on either computed tomography (CT) scan or nasal endoscopy [1]. The severity of CRSwNP disease is variable; frequently patients with a greater inflammatory disease burden often realize less benefit and earlier disease recurrence following medical and surgical therapy [2].

CRSwNP has been classified as a type 2 inflammatory disease in North America and Europe [3]. As such, following mucosal barrier dysfunction, immune dysregulation leads to the production of the key cytokines Interleukin (IL)-4, IL-5, and IL-13 and a sustained toxic inflammation which brings about the mucosal changes and the related disease symptomatology. Despite surgery and ongoing topical corticosteroid medical therapy, many patients remain symptomatic of CRSwNP and have incomplete disease control [4]. Monoclonal antibody therapy may provide a treatment option in this patient segment with poor disease control. Dupilumab, an anti-IL-13 and anti-IL-4Rα monoclonal antibody, recently demonstrated substantial CRSwNP disease modifying properties, which can lead to important symptom improvement for patients with difficult to control CRSwNP [5]. Dupilumab was first approved by Health Canada for use in patients with CRSwNP on August 18, 2020. This is the first monoclonal antibody therapy ever approved in Canada for the treatment of CRSwNP. As such, Otolaryngology-Head and Neck Surgeons in Canada caring for patients with CRSwNP have little experience using monoclonal antibody therapies in patient care.

Given the inexperience of clinicians managing patients with CRSwNP, the Canadian Rhinology Working Group produced a white paper providing guidance to treating physicians, both in Canada and internationally, when considering monoclonal antibody therapy for patients with CRSwNP [6]. Despite this guidance, questions about real-world efficacy of the processes for accessing treatment and treatment related outcomes in comparison to clinical trial results, are a concern for any new therapy [7]. The aim of this study was to provide our real-world experience of access to dupilumab therapy for patients with CRSwNP and disease-specific sinonasal outcomes for patients receiving dupilumab therapy for the indication of CRSwNP and to consider factors that may explain an efficacy-effectiveness gap, following the first year of drug access for patients treated in a tertiary care rhinology clinic.

## Patients and methods

### Data sources

A single center electronic medical record (EMR) practice audit of access to dupilumab therapy and disease-specific quality of life outcomes for patients with CRSwNP considered for dupilumab treatment was conducted. All identified patients had applied for dupilumab coverage through the FREEDOM program (Sanofi Genzyme, Mississauga, ON, Canada,). The result of this application was reviewed for each patient. Data on diagnosis, application status, and patient characteristics were obtained. The electronic medical record contains, among others, information on individual prescriptions with dupilumab dosage, date of first administration and administration duration as well as adverse events. Data was collected and managed in an excel spreadsheet (Microsoft, Seattle, WA, USA).

### Study population

Patients with CRSwNP who had undergone prior sinus surgery (if they did not have a medical contraindication to surgery) and had been managed with topical steroid therapy with or without systemic steroid were considered for dupilumab therapy between August 18, 2020 and Sep 15, 2021. Characteristics that were recorded for included patients were age (at visit for dupilumab application), gender, number of prior surgeries, time since last surgery, presence of asthma, NSAID sensitivity, aeroallergen sensitivity, start date of dupilumab therapy and number of doses of dupilumab up until the date of data cut off as well as adverse events.

## Reference outcome

The Sinonasal Outcome Test- 22 (SNOT-22) is a validated and reliable tool for assessing symptoms of chronic rhinosinusitis with polyps; its minimal clinically important difference (MCID) is 8.9 points [8]. At our centre, the SNOT-22 is routinely administered to patients to monitor the progress of therapies given to treat CRSwNP.

### Statistical analyses

We present a descriptive analysis of patient characteristics and the changes in SNOT-22 scores collected during the period of time of this chart review.

### Ethical statement

All methods were carried out in accordance with relevant guidelines and regulations. The study was approved by the research ethics board, (IRB #Pro00057336). The study was performed in accordance with the ethical standards of the 1964 Helsinki declaration and its later amendments or comparable ethical standards.

## Results

### Baseline characteristics

A total of 85 patients were considered for dupilumab therapy during the study period. Forty-three patients started therapy; ten patients are awaiting the decision from their insurer during the study period. We present the baseline data for the 53 patients who have started therapy or are awaiting a decision (Table 1). In this cohort, 24 were female; 38% of patients had an underlying diagnosis of Aspirin Exacerbated Respiratory Disease (AERD). The mean number of prior surgeries was 2.6 range [0–16], and the mean time since the last surgical procedure was 49.5 months. 49% of the cohort had a dust mite allergy. At the time of review, the mean number of dupilumab doses received by each patient was 23 [range 4–36]. The mean SNOT-22 score at baseline was 60.56 (SD 21.63), which is considered to indicate severe CRS disease symptomatology. At the time of application all patients were on a minimum of daily topical intranasal corticosteroid as a baseline therapy, specifically, 33 (62.3%) patients were using nasal saline irrigation mixed with budesonide while 20 (37.7%) patients were using a topical intranasal corticosteroid spray. Forty eight patients (90.56%) had a history of prior endoscopic sinus surgery (ESS), polypectomy or both.

**Table 1:** Patient characteristics

Age mean (SD)	52.94 (10)
Female Sex n (%)	24 (45.28)
AERD n (%)	20 (37.73)
Asthma n (%)	47 (88.67)
Dust Mite Sensitivity n (%)	26 (49.05)
Prior Sinus Surgery/Polypectomy n (%)	48 (90.56)
Endoscopic Sinus Surgery or in clinic polypectomy	46 (86.73)
Endoscopic Sinus Surgery and in clinic Polypectomy	2 (3.77)
Number of prior surgeries mean (range)	2.57 (0–16)
Time since last surgery to start of therapy—months (SD)	49.55 (34.21)
Baseline SNOT-22 score mean (SD)	60.56 (21.63)
Average number of doses received up to Jan 31, 2022 n(range)	23 (4–36)

AERD, Aspirin Exacerbated Respiratory Disease; SNOT-22, Sinonasal Outcome Test -22; SD, Standard deviation

### Insurer coverage for dupilumab

A total of 85 patients requested insurance coverage for dupilumab therapy between Aug 18, 2020 and Sep 15, 2021. Of these, 42 (49%) patients were able to attain coverage for the requested therapy; one patient was denied coverage but started therapy by paying out of pocket. The reasons for noncoverage of patients by their insurers are listed in Table 2. The most common reason was that the therapy was still considered to be under review by the insurer. At the time of the analysis of results for this manuscript, twelve months had passed since the approval of dupilumab therapy by Health Canada for patients with CRSwNP.

**Table 2:** Reasons dupilumab therapy was not started

Total submissions for coverage between Aug 18, 2020 and Sep 15, 2021	85
Insurance company did not consider coverage for dupilumab at the time of submission n (%)	23 (27)
Patient did not meet criteria for coverage	1 (1.17)
Other reasons therapy was not started	
Patient did not want to exhaust lifetime maximum limit of policy	1 (1.17)
Medical conditions not related to CRSwNP	3 (3.52)
Patient did not want to pursue therapy	5 (5.88)
Application still in process	10 (11.76)

CRSwNP, Chronic Rhinosinusitis with Nasal Polyps

### Outcomes with dupilumab therapy

**Fig. 1:**
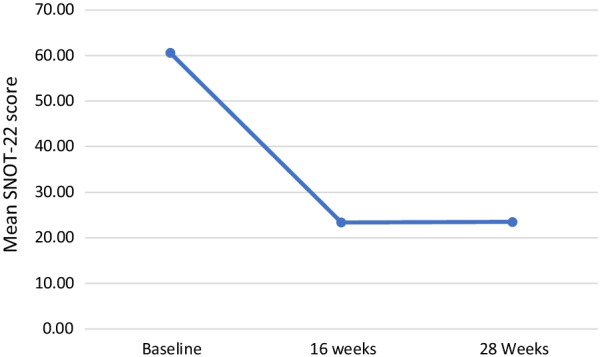
Mean SNOT-22 score in patients treated with dupilumab for CRSwNP

**Fig. 2:**
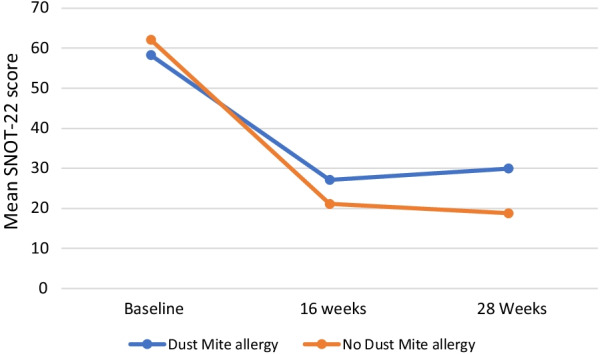
Mean SNOT-22 score in patients with and without dust mite allergy

All patients who initiated dupilumab therapy opted to receive therapy by self-injection at home rather than receiving treatment in the rhinology clinic. The cohort had a mean baseline SNOT-22 score of 60.56 (SD 21.63). After 16 weeks of treatment the mean SNOT-22 score decreased by 37 points to 23.36; this improvement was maintained at 28 weeks with a mean SNOT-22 of 23.47 (Fig. 1); sixteen patients had completed 1 year of therapy at the time of this review, the mean SNOT-22 score at this timepoint was 14.37 (SD 11.76). Thirty-three (64%) patients in this cohort did not have AERD; 27 had CRSwNP and asthma and 6 had CRSwNP alone. The mean baseline SNOT-22 of those without AERD was 57.44; at 16 weeks and 28 weeks the mean SNOT-22 scores were 22.28 and 21.36 respectively. Clinically, similar effects of dupilumab therapy on SNOT-22 scores were seen in patients with AERD. Mean SNOT-22 scores at baseline, 16 and 28 weeks in this group were 66.11, 25.46 and 26.78 respectively (Table 3). Baseline SNOT-22 scores were not clinically different in those with and without sensitivity to dust mites (58.25 vs 62.09). The 28 week mean SNOT-22 score was clinically higher in those with dust mite allergy, however (Fig. 2).

**Table 3:** SNOT-22 scores (SD) in patients receiving dupilumab therapy

	SNOT-22 Score		
	Baseline (n = 53)	16 weeks (n = 38)	28 Weeks (n = 36)
Full Cohort	60.56 (21.63)	23.36 (15.97)	23.47 (17.66)
CRSwNP	57.44 (20.86)	22.28 (15.32)	21.36 (16.36)
AERD	66.11(22.45)	25.46 (17.60)	26.78 (19.70)

SNOT-22, Sinonasal Outcome Test-22; SD, Standard Deviation; CRSwNP, Chronic Rhinosinusitis with Nasal Polyps; AERD, Aspirin Exacerbated Respiratory Disease

The mean baseline score of the loss of sense of smell and taste item of the SNOT-22 was 4.24 (SD 1.2), after 16 and 28 weeks of treatment, the mean score for this item was 1.8 (SD 1.7) and 1.65 (SD 1.53) respectively.

Baseline SNOT-22 scores grouped by domains showed that the rhinologic symptoms domain (maximum score = 30) had the highest score (mean 20.4 SD 5.8) followed by the sleep dysfunction domain (mean 15.6 SD 6.7) and the extra-nasal rhinologic symptoms domain (mean 8.7 SD 3.2). Table 4 shows the SNOT-22 scores stratified by domain for all time-points.

**Table 4:** SNOT-22 scores grouped by domains

	Baseline (n = 53)	16 weeks (n = 38)	28 weeks (n = 36)
Rhinologic Symptoms (maximum score = 30)	20.4 (5.85)	8.07 (4.1)	7.52 (4.85)
Extra-Nasal Rhinologic Symptoms (maximum score = 15)	8.76 (3.24)	3.07 (2.4)	2.72 (2.04)
Ear/Facial Symptoms (maximum score = 25)	9.24 (5.72)	3.73 (2.9)	3.97 (4.06)
Psychological Dysfunction (maximum score = 35)	18.58 (8.75)	6.84 (7.71)	7.34 (7.63)
Sleep Dysfunction (maximum score = 25)	15.67 (6.72)	6.57 (6.28)	7.02 (7.26)

### Adverse events related to dupilumab therapy

At each follow-up appointment patients were screened for the following potential adverse events: changes in their asthma control, arthralgia, ocular changes (e.g. conjunctivitis, dryness, eye irritation), local reactions at the injection site, dermatologic changes, nasopharyngitis). Each patient was also offered the opportunity to inform their clinician of any new symptoms they may be experiencing since the initiation of dupilumab therapy. One patient reported a single incident of transient skin erythema at the injection site lasting up to 2 h. A second patient reported experiencing dry eyes but the patient has a history of thyroid eye disease which can also be associated with dry eyes. No therapy was sought for this symptom. A third patient reported bilateral joint pain specifically in the knees and ankles that started 3 months after the start of dupilumab therapy. At this time dupilumab was stopped and the patient was referred to a rheumatology specialist. A full rheumatologic panel was done and was negative. Patient resumed dupilumab therapy after 4 months but reduced the frequency of injection to once a month instead of every two weeks. At this time the patient noticed a slight return of joint pain and stiffness but no swelling. A fourth patient reported ongoing cough that required prednisone as determined and prescribed by their family physician. Lastly, one patient reported having been hospitalized for 4 days due to an episode of asthma exacerbation while on dupilumab therapy.

## Discussion

We present our experience using dupilumab for the treatment of CRSwNP in the first year since it was approved in Canada for this indication. Improvement in the SNOT-22 scores seen in our cohort after 28 weeks (mean SNOT-22 = 23.47 SD 17.66; mean score change from baseline = − 32.85 SD 21.10) of therapy exceeded the minimal clinically important difference of 8.9 points. These changes were also seen in the efficacy of dupilumab in clinical trials (SNOT-22 score at 24 weeks for the treatment group in the SINUS-52 study was 23.84, with a least squares mean change from baseline of − 27.77) [5] and although a direct comparison cannot be made due to our small sample size, the results in our cohort show that similar clinical patient reported symptom improvement may be achieved outside of the controlled environment of clinical trials.

In the last 12 months at our centre, 85 patients applied for insurance coverage for dupilumab therapy, 42 patients (49%) received coverage and started therapy; one additional patient was denied coverage but started therapy by paying out of pocket. The main reason given by the insurers was that they were not considering coverage for the CRSwNP indication at the time of application. To date, the Canadian Agency for Drugs and Technologies in Health (CADTH) has provided a reimbursement recommendation for dupilumab for the atopic dermatitis and asthma indications [9, 10], however a reimbursement review has not been completed for the CRSwNP indication.

This study sought to identify evidence, from a single center tertiary care rhinology practice, of an efficacy-effectiveness gap between clinical trial results with dupilumab in comparison to real world experience. Baseline mean SNOT-22 score of the practice cohort (60.56, SD 21.63) exceeded the MCID when compared to the baseline data of the dupilumab trial groups (50.94, SD 20.66). However, the 24-week SNOT-22 scores (23.89, SD 18.77) from the treatment group in the SINUS-52 clinical trial were not different than similar time SNOT-22 scores of this clinical cohort (23.36 SD 15.97). This suggests that there does not appear to be an efficacy-effectiveness gap when considering symptom improvement as reported by patients using the SNOT-22 questionnaire. We were unable to evaluate other outcomes included in the SINUS- 24 and SINUS 52 studies such as polyp score, olfactory function using objective measures and Lund-Mackay CT scores, however, the SNOT-22 is a well validated instrument that is able to discern treatment effects and is representative of patient CRS-related quality of life. Further, the SNOT-22, a subjective patient reported outcome measure (PROM), has been identified as a key CRS outcome for research and currently the most notable when considering patient preference and value judgements for treatment options [11, 12]. As a yet even greater range of patients are prescribed dupilumab therapy, an efficacy-effectiveness gap could become evident due to factors such as comorbidities, compliance with both dupilumab and baseline medical therapy, and the severity of the disease, amongst other factors.

The Canadian Rhinology Working Group recommends that biologic therapies for chronic rhinosinusitis be considered only for patients who have failed medical therapy and have undergone sufficient sinus surgery or for patients who cannot undergo sinus surgery and have failed medical therapy [6]. However, at this time, access to dupilumab for Canadian patients and specifically in the province of Ontario, remains limited to only a fraction of those with private insurance. Currently, patients without private insurance have few options for attaining treatment with any monoclonal antibody therapy. For most patients, the therapy is cost prohibitive with only one patient from the current study having the means to self-fund therapy.

Long-term follow-up data is needed in order to determine if the higher yearly cost of dupilumab therapy is offset by the need for multiple surgeries, disease complications, the costs and any adverse effects of systemic and topical corticosteroids as well as the impact on the quality of life for patients who have poorly controlled CRSwNP. Underappreciated, is the effect of CRSwNP on the olfactory ability of patients, with those affected having levels of impact ranging from impairment, to disability and potential handicap [13]. In particular, the economic burden due to the sensory disability and, for some patients, the resultant handicap from olfactory loss has yet to be fully evaluated. Furthermore, anosmia presents a major safety issue for the anosmic individual and any of their dependents. CRSwNP patients treated with dupilumab experience improvement in their olfactory function [5]. In the present study this improvement was evidenced by a decrease in the SNOT-22 smell/taste score, however, the long-term effects of dupilumab on olfactory function require further study.

## Conclusion

As with any new therapy, but specifically with the advent of biologic therapy for CRS, clinicians will rightfully be seeking to evaluate an efficacy-effectiveness difference between the trial results and clinical practice. The current study, although small, provides a first look for this difference. No efficacy-effectiveness gap is evident from the current results. Further study is required to evaluate the long-term implications of monoclonal antibody therapy use in patients with poorly controlled CRS despite adequate surgical and medical therapy.

## Data Availability

The datasets used and/or analysed during the current study are available from the corresponding author on reasonable request.
